# Influence of calcium carbonate sludge on cement-stabilized subgrade quality as investigated by means of electrical resistivity measurements

**DOI:** 10.1038/s41598-023-46282-x

**Published:** 2023-11-08

**Authors:** Narongchai Wiwattanachang, Chanarop Vichalai, Pham Huy Giao

**Affiliations:** 1 Department of Civil Engineering, Faculty of Engineering and Architecture, Rajamangala University of Technology Suvarnabhumi (RUS), 450 Suphanburi-Chainat Rd, Suphanburi, 72130 Thailand; 2 Asian Institute of Technology (AIT), Khlong Nueng, Thailand; 3 PetroVietnam University (PVU), Vũng Tàu, Vietnam

**Keywords:** Engineering, Materials science

## Abstract

Calcium carbonate [CaCO_3_] is a key raw material used in the clarification of sugarcane juice for syrup production. The CaCO_3_ sludge produced during the clarification process is waste that needs to be stored, creating a geoenvironmental problem. On the other hand, it has been found that cement-stabilized subgrade is a suitable alternative for improving the quality of a subgrade course. This study aimed to investigate the influence of calcium carbonate sludge on the quality of the subgrade. The subgrade was composed of a mixture of 10% to 30% CaCO_3_ sludge, 1% to 3% of original Portland cement (OPC), and 67% to 100% of unqualified crushed rocks by weight. The modified Proctor method was used to compact soil–cement admixture samples, which were then tested for mechanical properties and electrical resistivity. The Wenner electrode array was used to measure electrical resistivity and compare it to the unconfined compressive strength of 16 different types of soil–cement mixtures after 7 days. The results of experiments show that the basic properties of CaCO_3_ sludge, when mixed with OPC and packed down, can make the best soil–cement mixture. As a result of this study, electrical resistivity was found to be in good correlation with unconfined compressive strength, thus opening up a time-saving and cost-effective way to check the quality of a soil–cement mixture.

## Introduction

Cement-stabilized subgrade (CSS) is a compacted, engineered mixture of pulverized in-situ soil, water, and moderate proportions of cement that result in a semi-bound to bound material. The benefits of CSS include improved shear and compressive strength, as well as reduced soil shrinkage and swelling tendencies. The volume of cement used and the type of soil are factors that determine the level of improvement^1^.

Lime and original Portland cement (OPC) can be used for ground improvement and high-grade road construction^2–7^. The OPC has been used to improve the quality of the soil–cement mixture^8–13^. Cement treatment can help to maintain project timelines and minimize the impacts of gap-graded soil on pavement design^1^.

**Figure 1:**
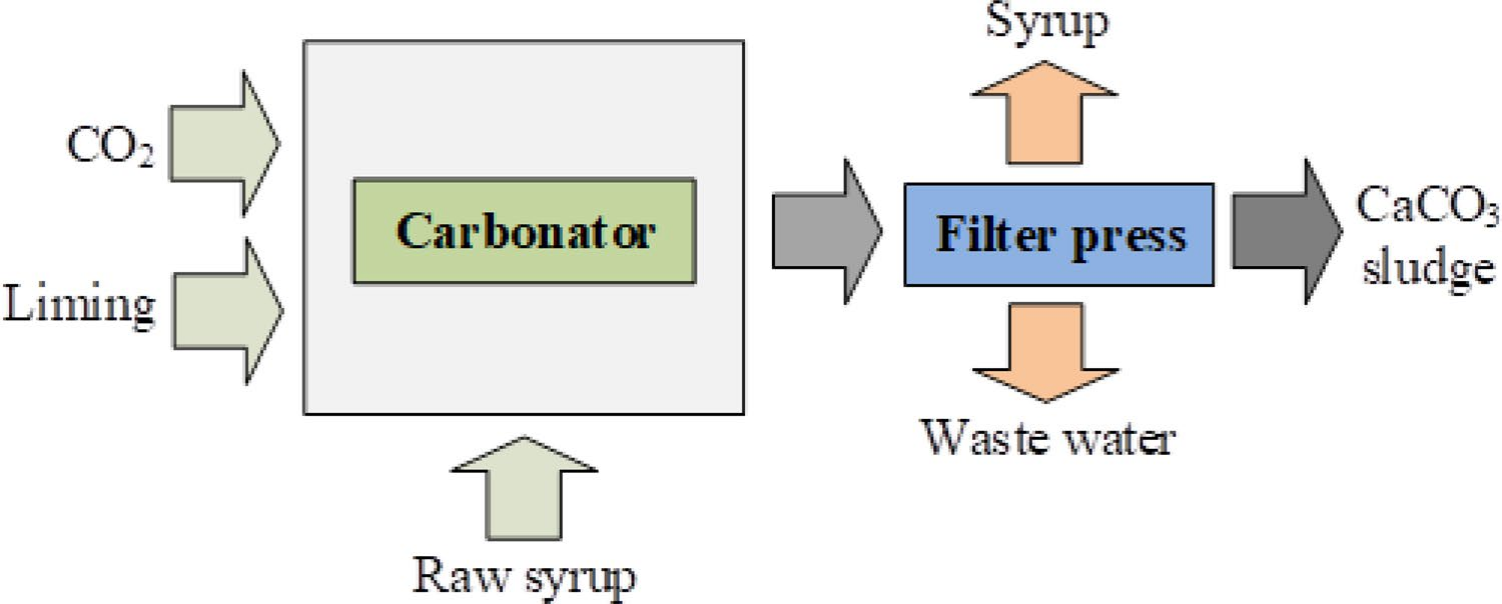
Clarification of sugarcane juice for syrup production.

**Figure 2:**
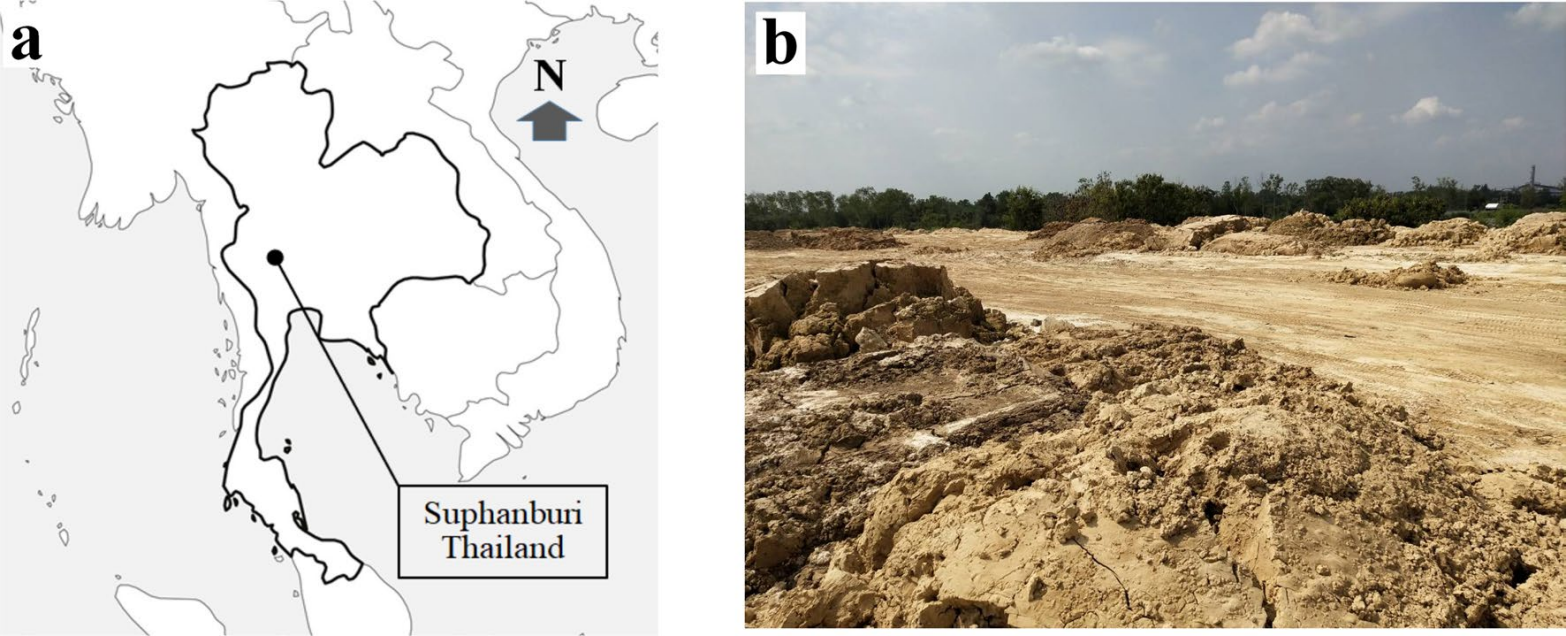
Location of the study site. (**a**) Suphan-Buri province of Thailand. (**b**) Calcium carbonate sludge storage area.

Calcium carbonate [CaCO_3_] is a key raw material used in the clarification of sugarcane juice for syrup production^14^, as shown in Fig. 1. The study was conducted in the Suphan-Buri province of Thailand, as indicated in Fig. 2 (left-hand side inset), which also shows an area where CaCO_3_ sludge is stored after the clarification process (Fig. 2, right-hand side inset). The clarification process is necessary to reduce particles in sugarcane juice before heating to produce syrup^15^. Although CaCO_3_ is insoluble in water^16^, it can react with water and carbon dioxide [CO_2_] to form calcium bicarbonate [Ca(HCO_3_)_2_].

The effect of nano-CaCO_3_ on the ultimate compressive strength of the soil in the XRD patterns indicated an increase in the recrystallization of the particles^17^. The marine environment had a great influence on the strength and durability of cement-stabilized soil, and compound nano-CaCO_3_ addition could effectively improve the compressive strength of cement-stabilized soil at early and late ages due to the nucleation and nano-filling function^18^. CaCO_3_ has also been widely mixed with Portland cement in research to demonstrate its impact on concrete quality^19–22^.

Giao et al.^23^ conducted a study on the geotechnical properties of clay deposits in Busan, South Korea, using electrical resistivity measurements. The study involved over 50 clay samples collected from various locations worldwide and was able to establish a correlation between electrical resistivity and other geotechnical parameters such as salinity, water content, unit weight, and sampling depth. Similar studies were performed by Liu et al.^24^ Bery and Saad^25^ Zhang et al.^26^. Some studies have found a relationship between electrical resistivity and hydraulic conductivity for certain soil types, e.g., Abu-Hassanein et al.^27^, Latt and Giao^28^.

Liu et al.^29^ investigated the relationship between electrical resistivity and curing time of soil–cement admixtures, as well as the influence of OPC volume on unconfined compression strength (UCS). They found that the UCS is proportional to the increased OPC volumes. However, it is important to note that the finite size of the samples tested in the experiment can affect the electrical resistivity measurements, which have to be corrected for geometric effects depending on the measuring setup^30,31^.

The goal of this study is to find out if adding CaCO_3_ sludge to a mix of OPC and unqualified crushed rocks will improve the quality of the subgrade course in terms of unconfined compressive strength (UCS). This study supports the waste-to-wealth approach by reusing CaCO_3_ sludge, which causes environmental problems such as particulate matter with a diameter of less than 2.5 microns and wastewater. In addition, using the cement treatment with CaCO_3_ sludge can improve soil cement properties and reduce compaction energy. The CaCO_3_ sludge improves the optimum moisture content (OMC) under different humidity conditions.

The influence of CaCO_3_ sludge in the admixture will be assessed through resistivity measurements. The estimation guidelines for OPC and CaCO_3_ sludge dosage will be used for subgrade courses to obtain mix proportions that meet engineering requirements. Also, we want to find a link between electrical resistivity and UCS so that electrical resistivity can be used as a quick way to measure UCS and the overall quality of the subgrade mixed with CaCO_3_ sludge.

## Experimental program

### Materials

Materials used in the experiment include OPC, CaCO_3_ sludge, and unqualified crushed rocks with properties shown in Table 1.

**Table 1:** Material characteristics.

Material	Sample type	Characteristics
OPC	Type-*I*	Specific gravity = 3.16
CaCO_3_ sludge	Oxide powder	1–10 µm
Unqualified crushed rocks	Well grade	The maximum size = 45 mm and pass sieve No.200 = 20% Liquid Limit (LL) = 16%, Plasticity Index (PI) = 12
Water	Tap water	pH = 7

Table 2 shows the results of the semi-qualitative analysis of OPC and CaCO_3_ sludge's chemical composition. The main oxide found in both materials is CaO, which amounts to 63.82% and 97.24% in OPC and CaCO_3_ sludge, respectively. Other components found in OPC include MgO, Al_2_O_3_, SiO_2_, P_2_O_5_, SO_3_, K_2_O, MnO, and Fe_2_O_3_, and loss on ignition (LOI) was followed by ASTM C114-11b^32^, while CaCO_3_ sludge also contains small amounts of MgO, Al_2_O_3_, SiO_2_, P_2_O_5_, SO_3_, K_2_O, Fe_2_O_3_, SrO, and LOI. These results can be useful for understanding the properties of these materials and their potential applications. CaO is an important factor in the main compound in OPC when calculated by Bogue's equation ^33^.

**Table 2:** Chemical composition by semi-qualitative analysis.

Chemical analysis	OPC (wt%)	CaCO_3_ sludge (wt%)
MGO	2.35	0.51
Al_2_O_3_	5.56	0.13
SiO_2_	20.64	0.77
P_2_O_5_	–	0.09
SO_3_	1.88	1.04
K_2_O	0.95	0.01
CaO	63.82	97.24
MnO	–	0.02
Fe_2_O_3_	3.25	0.16
SrO	–	0.03
LOI	1.55	–

3.

**Figure 3:**
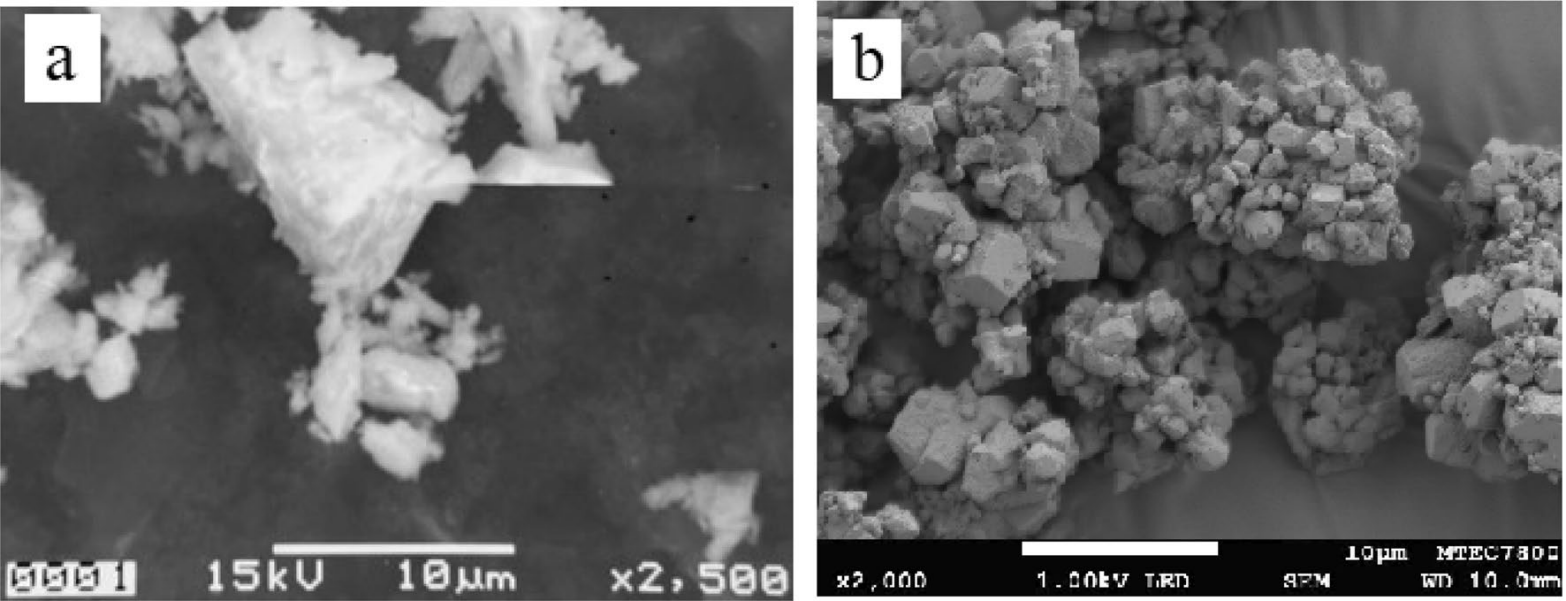
SEM images of a mixture with (**a**) original Portland cement (OPC) and (**b**) calcium carbonate [CaCO_3_] sludge.

Figure 3 presents the results of the scanning electron microscope (SEM) analysis of the particles of OPC and CaCO_3_ sludge. Figure 3a shows that the particles of OPC are deformed and have an amorphous structure. This is due to the rapid cooling and grinding processes, which can result in the formation of amorphous particles. The amorphous particles have an irregular structure and lack a well-defined crystalline structure.

Figure 3b shows the particles of the CaCO_3_ sludge, which are coagulated, smooth, and angular in shape. The coagulation of particles is due to the agglomeration of small particles into larger ones. The smooth and angular shape of the particles indicates that they are well-formed and have not undergone any significant alteration in shape or size during processing. Overall, the SEM analysis provides valuable information about the characteristics of the particles of the OPC and CaCO_3_ sludge, including their morphology, texture, and structure.

### Mix design proportion

Table 3 shows the mix design proportions, consisting of a CaCO_3_ sludge ratio of 10% to 30%, an OPC of 1% to 3%, and unqualified crushed rocks in a ratio of 67% to 100% by weight. The water content of soil–cement admixtures is determined using the water content at the optimum moisture content, as tested according to ASTM D1557.

**Table 3:** Soil–cement admixture proportion.

Mix	OPC (%)	CaCO_3_ sludge (%)	Unqualified crushed rocks (%)
CONTROL	0	0	100
SC_1_-0	1	0	99
SC_2_-0	2	0	98
SC_3_-0	3	0	97
SC_0_-10	0	10	90
SC_1_-10	1	10	89
SC_2_-10	2	10	88
SC_3_-10	3	10	87
SC_0_-20	0	20	80
SC_1_-20	1	20	79
SC_2_-20	2	20	78
SC_3_-20	3	20	77
SC_0_-30	0	30	70
SC_1_-30	1	30	69
SC_2_-30	2	30	68
SC_3_-30	3	30	67

### Experimental program

#### Soil–cement casting and testing

The optimum moisture content (OMC) of soil–cement admixtures is determined by the modified Proctor compaction test followed by ASTM D1557, as mentioned above. UCS is tested using the following procedure: (i) place the sample stick in the center of the lower press pedal, moving the upper keypad to touch the sample properly; (ii) adjust the gauge for measuring contraction and force to be located at the center; and (iii) press the sample with a vertical rate of movement and strain rate in the range of 0.5% to 2% per minute.

#### Electrical resistivity testing

**Figure 4:**
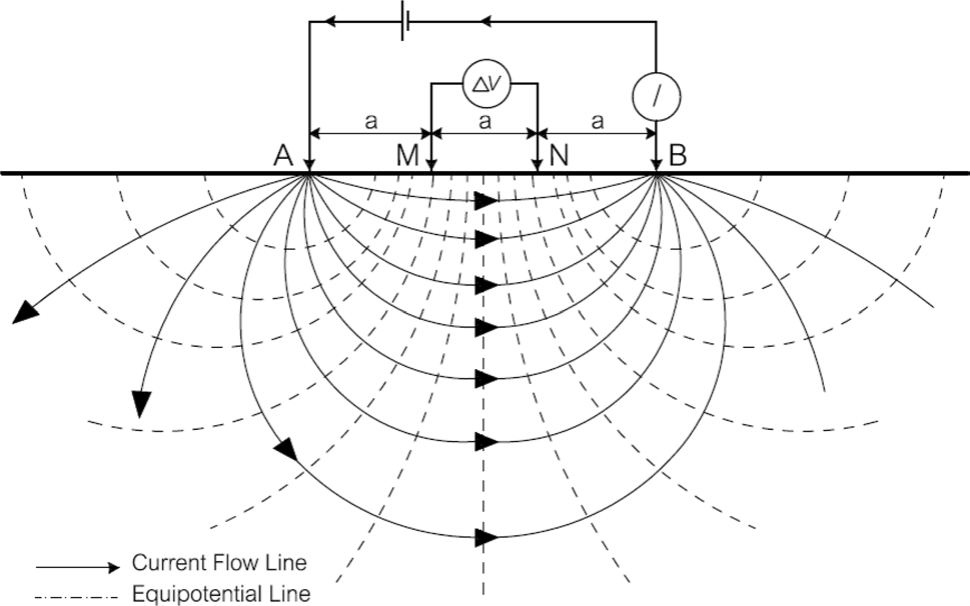
Wenner arrangement of electrodes and distribution of electric field underneath^31^.

Figure 4 shows the Wenner electrode array, which is commonly used in field resistivity surveys. The array consists of four equally spaced electrodes, including a pair of current electrodes (*A* and *B*) through which electric current is injected into the ground and a pair of potential electrodes (*M* and *N*) across which the difference in electric potential is measured.

The apparent electrical resistivity (*ρ*_a_) in Ω m can be determined based on the current intensity (*I*) in mA and the potential difference between *M* and *N* (Δ*V*) in mV readings using the following formula:

1 $$ \rho_{a} = k\frac{\Delta V}{I} $$

Here, *k* is called the geometric factor and is defined by Eq. (2) below. The value of *k* in the formula for calculating the apparent electrical resistivity depends on the geometry of the electrode array used. For the Wenner array, the geometric factor is defined as *k* = 2*πa*, where *a* is the distance between adjacent electrodes.

2 $$ k = \frac{2\pi }{{\left( {\frac{1}{AM} - \frac{1}{BM} - \frac{1}{AN} + \frac{1}{BN}} \right)}} $$

When measuring electrical resistivity on laboratory core samples, the finite size of the sample can affect the accuracy of the measurements, and it is necessary to correct for this geometric effect. Morris et al.^30^ proposed a correction factor (*K*_c_) to adjust the measured (or apparent) resistivity (*ρ*_a_) of a cylindrical concrete sample of limited size. Other studies, such as Giao et al.^23^ and James and Pandian^2^, have also explored this issue in more detail.

**Figure 5:**
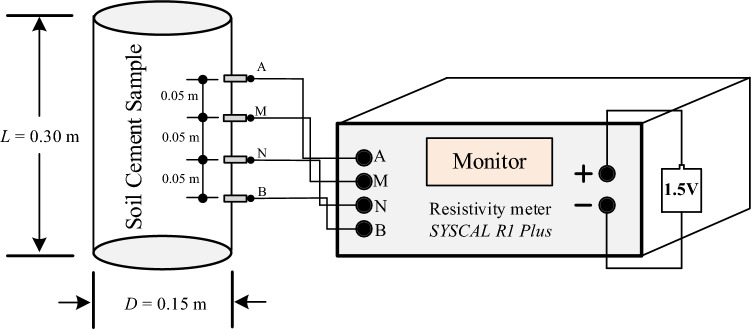
Testing setup to measure the resistivity on a soil–cement admixed sample.

The setup for measuring the electrical resistivity of soil–cement admixtures used in this study is based on the design proposed by Giao et al.^23^. It uses four steel needle electrodes with a diameter of 6 mm and a length of 30 mm, which are inserted into the sample to a depth of 10 mm. The electrodes are spaced 0.05 m apart from each other and arranged in a Wenner array configuration (as shown in Fig. 5). The soil–cement admixture is placed in a PVC mold with a diameter of 0.15 m and a length of 0.30 m.

The silicone sealant is used to fill the gap formed between the probe and PVC. For the upper and lower ends of the soil, the cement sample is closed with a plastic sheet to prevent moisture from evaporating into the outside atmosphere. To control moisture during the hydration reaction, the sample is cured using a chemical curing process according to ASTM C 309 standards. The electrical resistivity measurements are taken using this setup on the cured soil–cement admixture samples.

## Results and discussions

### Maximum dry density, MDD

**Figure 6:**
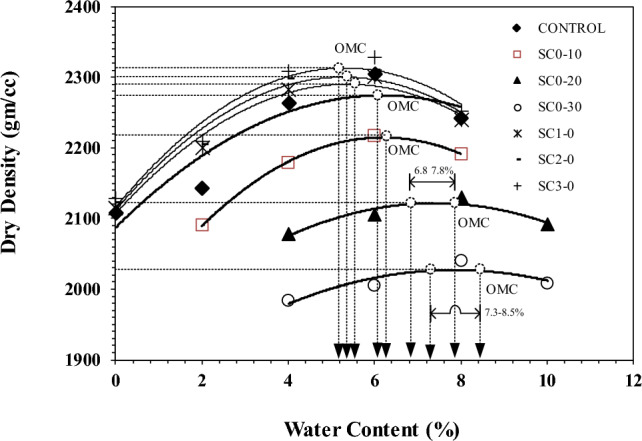
Modified compaction curves show the optimum moisture content (OMC) for different OPC samples with and without CaCO_3_ (refer to Table 3).

The impact of using CaCO_3_ sludge as a replacement for unqualified crushed rocks in ratios of 10%, 20%, and 30%, respectively, compared to CONTROL (see Table 3), can be seen in Fig. 6. According to measurements made using modified Proctor tests, the figure demonstrates that the higher the percentage of CaCO_3_ sludge, the lower the dry density and the higher the water content.

For SC_1_-0, SC_2_-0, and SC_3_-0, which used Portland cement to replace unqualified crushed rocks of 1%–3%, the maximum dry density was between 2,290 and 2,315 gm/cc and the water content was 5.2%–5.6%. According to the findings, MDD increases with cement content. There were slight increases in OMC but minor decreases in MDD.

Table 4 compares the MDD and OMC values from this study to those from previous studies. Chummuneerat et al.^34^ found that crushed rock containing 2% OPC had OMC and MDD values of 6.3% and 2.327 gm/cc, respectively. These findings are consistent with those of Djellali et al.^35^ and Okonkwo et al.^36^. The higher Portland cement content is inversely proportional to the water content because Portland cement sucks water to perform hydration reactions in the bonding process. The specific gravity of cement particles is greater than that of unqualified crushed rocks. The addition of cement to a particular percentage of unqualified crushed rocks raises the density of the entire mass^35^.

**Table 4:** Comparing the values of MDD and OMC in this study with those from some previous studies.

Authors/samples	0% OPC		1.0% OPC		2.0% OPC		3.0% OPC	
	MDD (gm/cc)	OMC (%)	MDD (gm/cc)	OMC (%)	MDD (gm/cc)	OMC (%)	MDD (gm/cc)	OMC (%)
This study	2,275	6.1	2,290	5.6	2,300	5.4	2,315	5.2
Chummuneerat et al.^34^	2,310	5.8	–	–	2,327	6.3	–	–
Djellali et al.^35^	2,230	7.1	–	–	2,260	6.9	–	–
Okonkwo et al.^36^	2,020	8.3	–	–	–	–	2,125	5.8

Specifically, for SC_0_-10, SC_0_-20, and SC_0_-30, the maximum dry density is 3%, 7%, and 11% lower than the CONTROL, respectively. The CaCO_3_ sludge provides a wider range of optimum moisture content (OMC) as the CaCO_3_ sludge increases. For example, SC_0_-20 has an OMC width range of 1%, ranging from 6.8% to 7.8%, while SC_0_-30 has an OMC width range of 1.2%, ranging from 7.3% to 8.5%.

It shows that CaCO_3_ sludge is a material that improves quality and stability under OMC for subgrade. The reason for this phenomenon can be attributed to the tiny particle size and high water-absorbing characteristics of CaCO_3_ sludge, as shown in Fig. 5a. The filler effect of fine CaCO_3_ particles ensures the optimum moisture content (OMC) of soil compaction, which increases cohesiveness and uniformity.

### Unconfined compressive strength, UCS

**Figure 7:**
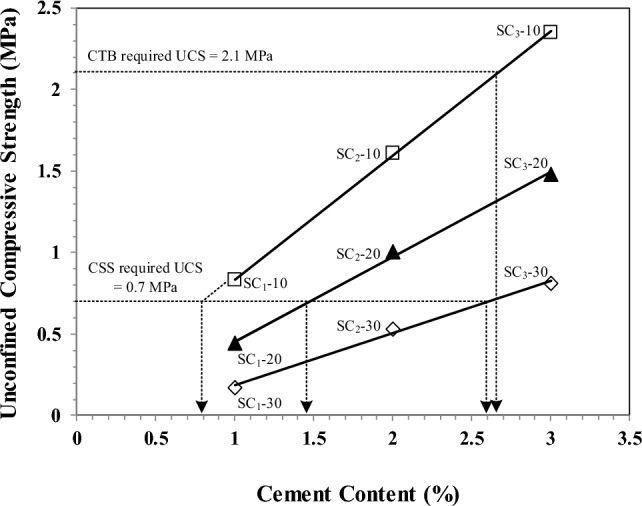
Empirical relationship between cement content (1%–3%), CaCO_3_ sludge content, and unconfined compressive strength (UCS) for cement-treated base (CTB) mixture.

The results of an unconfined compressive strength (UCS) trial of soil–cement samples in Table 3 showed that UCS increases with increasing cement content and decreasing CaCO_3_ sludge content, as shown in Fig. 7. The cement content promotes hydration reactions in different ratios, and the soil–cement mixture forms a solid mass with a higher unconfined compressive strength^37^.

Calcium silicate hydrate, an essential component of the hardened paste that provides cement-based structures with strength, can be created by the reaction of calcium carbonate sludge with Portland cement. The reaction is described as follows:

3 $$ {\text{CaCO}}_{{3}} + {\text{ 2SiO}}_{{2}} + {\text{ 6H}}_{{2}} {\text{O }} - > {\text{ Ca}}^{{{2} + }} + {\text{ CO}}^{{{32} - }} + {\text{ 2Si}}\left( {{\text{OH}}} \right)_{{4}} + {\text{ 4H}}^{ + } $$

The minimum UCS requirements for cement-stabilized subgrade (CSS) trials were above 0.70 MPa (100 psi) at a 7-day curing time^1^. The trials found that the cement content was 0.80, 1.48, and 2.60 percent, and the CaCO_3_ sludge ratios were 10, 20, and 30 percent by weight, respectively. The main target of this study is not to get a higher UCS but to achieve the required standard UCS for pavement with less OPC. As shown in Fig. 7, for the required standard USC value of 0.7 MPa, we can have different mixtures, i.e.: (i) SC_1_-10: with 10% CaCO_3_ sludge and 0.8% of OPC; (ii) SC_1_-20: with 20% CaCO_3_ sludge and 1.48% of OPC; and (iii) SC_1_-30: with 30% CaCO_3_ sludge and 2.6% of OPC, among which the mixture of SC_1_-10 has a clear advantage in having less OPC but still meets the requirement of UCS.

The cement-treated base (CTB) is calculated for UCS of not less than 2.10 MPa or 300 psi^1^. The OPC should be used at a ratio of 2.68 percent versus a CaCO_3_ sludge of 10 percent (Fig. 7). According to the results of the experiment, the maximum UCS of CaCO_3_ sludge at an OPC 3% mixture ranging from 20 to 30% was 1.45 and 0.80 MPa, respectively. In order to achieve the ultimate compressive strength (UCS) of 2.10 MPa or 300 psi required by the CTB, the experimental findings propose utilizing a mixture consisting of 2.68 percent ordinary Portland cement (OPC) and a maximum of 10 percent calcium carbonate (CaCO_3_) in the sludge.

Calcium silicate hydrate, a gel-like substance that fills the pores and gaps in the cement paste, is created when the calcium ions in calcium carbonate sludge mix with the silicate ions in Portland cement^38^. When calcium carbonate sludge is added to cement-stabilized subgrade quality, the mixture's workability and optimal moisture content (OMC) can be improved under high humidity conditions, opening up the possibility of low-cost construction. Therefore, it is important to carefully consider the amount of CaCO_3_ sludge used in CTB mixtures and ensure that the mixture meets the required UCS specifications.

**Figure 8:**
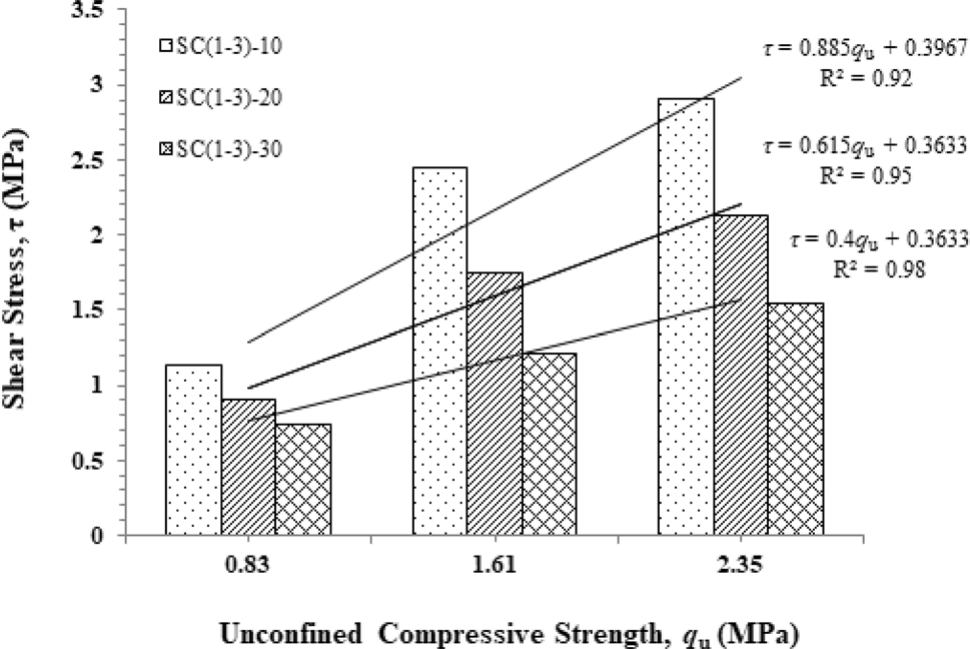
Shear stress *τ* against and unconfined compressive strength *q*_u_.

Figure 8 shows the relationship between shear stress and unconfined compressive strength. The study divided the sample into three groups: 10%, 20%, and 30% CaCO_3_ sludge, with 1%–3% Portland cement in each group. It has been noted that the shear strength envelopes exhibit linear behavior with respect to net normal stress.

Cement content is the most influential factor in determining maximum shear stress and UCS. The SC_(1–3)_-10 requires CaCO_3_ sludge to have a fixed rate of 10% and increases OPC from 1 to 3%, revealing that linear equations have a steeper slope than the SC_(1–3)_-20 and SC_(1–3)_-30, which are composed of CaCO_3_ sludge in the proportions of 20%–30%. The results indicate that increasing OPC doses at a 10% CaCO_3_ sludge concentration will have a higher effect on shear stress than UCS. On the other hand, as the quantity of CaCO_3_ sludge increases, the linear curve will have a shallower slope, indicating that it will have a greater effect on UCS than shear stress. However, previous studies have indicated that Portland cement improves soil–cement quality, as demonstrated by Horpibulsuk et al.^8^, Nguyen et al.^39^.

Shear modulus of soil (*G*_M_^)^ can be calculated based on the elastic modulus (*E*_s_) and Poisson's ratio (*υ*) as shown in Eq. (4):

4 $$ G_{M} = \frac{{E_{s} }}{{2\left( {1 + \nu } \right)}} $$

**Figure 9:**
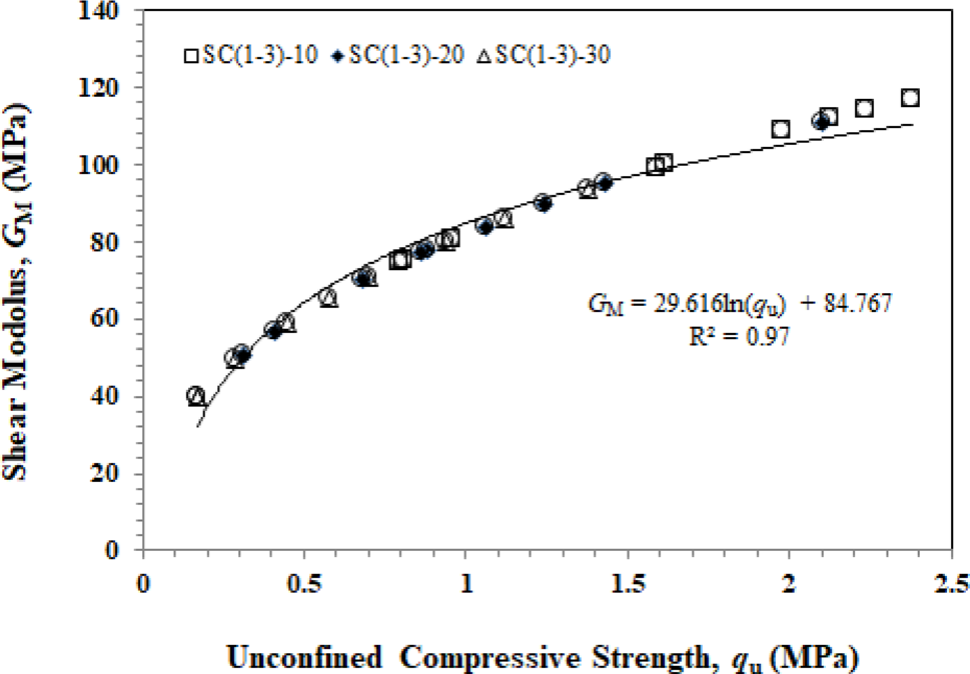
Relationship between the shear modulus and unconfined compressive strength.

The relationship between shear modulus and unconfined compressive strength is plotted in Fig. 9, which is in agreement with some previous studies that found *G*_M_ increases with UCS (*q*_u_) and cement content^40,41^. Shear modulus ranges from 39.6 to 116.8 MPa for SC_1_-30 and SC_3_-10, while unconfined compressive strength ranges from 0.17 MPa at the lowest to 2.38 MPa. The correlation between the shear modulus and unconfined compressive strength is very good with R^1^ equal to 0.97 as shown in Fig. 9.

### Relationship between the electrical resistivity and UCS

**Figure 10:**
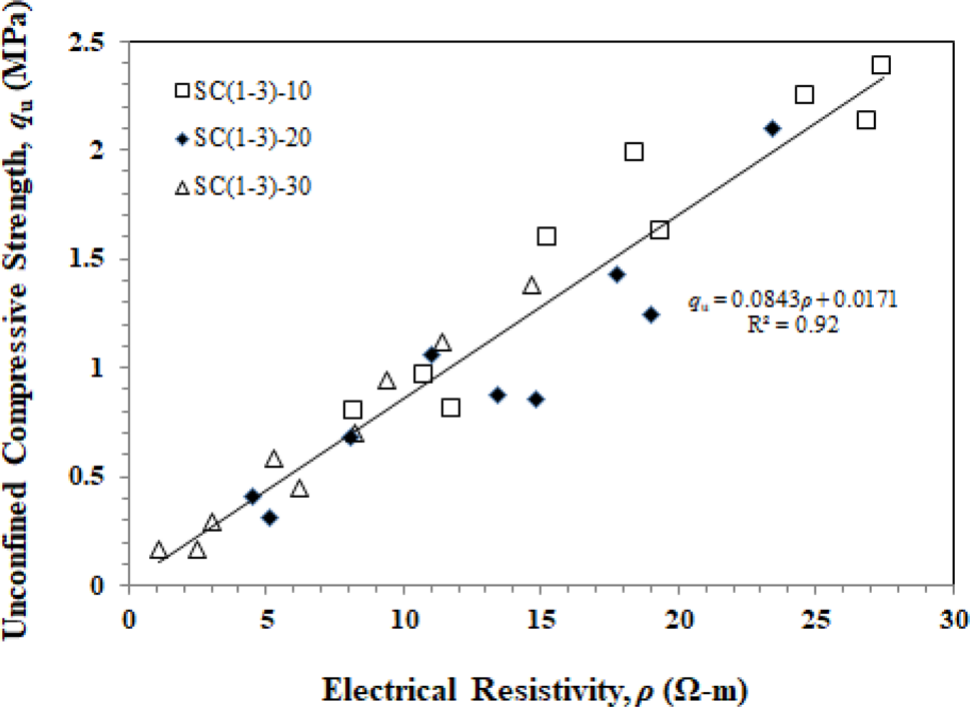
Unconfined compressive strength vs electrical resistivity of soil–cement admixtures.

Measurement of resistivity soil–cement samples at a 7-day curing time with a Wenner electrode array and using Morris's correction (*K*_c_)^30^ compared with UCS as shown in Fig. 10. The results of the experiment showed that the relationship between electrical resistivity (*ρ*) and unconfined compressive strength (*q*_u_) is proportional to unconfined compressive strength. The presence of OPC in ingredients is a crucial element in electrical resistance^42,43^.

The results demonstrated that the electrical resistivity of cement-treated soils increases as cement content rises^44^. While the increased amount of CaCO_3_ sludge content is inversely proportional to electrical resistivity, A correlation between these two parameters was obtained, as shown in Eq. (5):

5 $$ q_{u} = 0.0843\rho + 0.0171 $$

$$ R^{2} = 0.92 $$

The correlation coefficient *R*^2^ = 0.92 demonstrates a strong correlation between soil–cement resistivity and the unconfined compressive strength of the material. When compared to Liu et al.^24^ and Zhang et al.^44^, the results of this experiment indicated a similar tendency.

**Figure 11:**
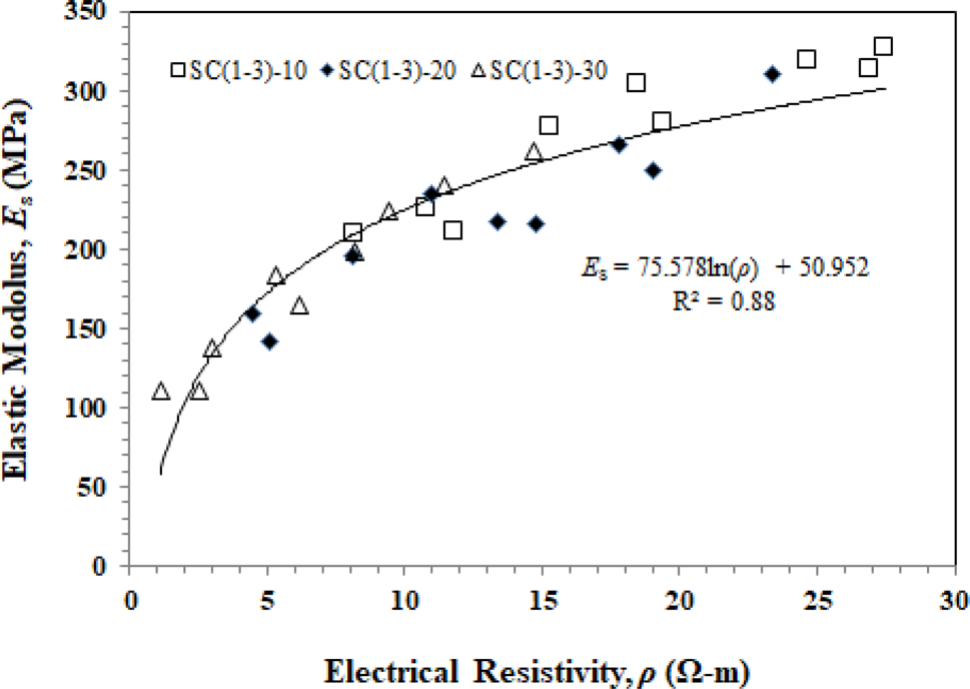
Relationship between the elastic modulus and electrical resistivity of soil–cement samples mixed with CaCO_3_ sludge.

As was already indicated, OPC is the main reason for the greater mechanical and electrical resistivity of soil–cement. The relationship between elastic modulus and electrical resistivity is seen in Fig. 11. When compared to UCS, elastic modulus characteristics are determined by cement usage, soil type, and chemical additives^45^. These two parameters were found to be correlated, as given in Eq. (6):

6 $$ E_{s} = 75.578\ln \left( \rho \right) + 50.592 $$

$$ R^{2} = 0.88 $$

This indicates that soil–cement resistivity can be effectively utilized to monitor the strength (*q*_u_) and elastic modulus (*E*_s_) of the material. The advantages of measuring electrical resistivity in soil and cement include the following: (i) It is a non-destructive test that allows real-time monitoring of the material; (ii) Resistivity measurement is reliable and quick, resulting in cost savings.

## Conclusions

The use of CaCO_3_ sludge in cement-stabilized subgrade quality was investigated through aggregate testing and electrical resistivity measurements, and the following conclusions can be drawn:

1. The addition of CaCO_3_ sludge results in a decrease in dry density, while the water content tends to increase. Therefore, the water content influences dry density to decrease when the CaCO_3_ sludge content exceeds 20%.
2. Unconfined compressive strength increases proportionally with the amount of cement content and decreases inversely with the CaCO_3_ sludge content.
3. This study suggests a mix of CaCO_3_ sludge, OPC, and unqualified crushed rocks to improve the quality of the subgrade course and meet the needs of the project.
4. The shear moduls of soil (*G*_M_) was found in a good relrionship with the unconfined compressive strength as shown in Fig. 9 with a correlation coefficient *R*^2^ = 0.9248.
5. To predict unconfined compressive strength by measuring electrical resistivity, simple equations can describe the relationship between electrical resistivity and mechanical properties of soil–cement admixtures.
6. The electrical resistivity measurement method is a quick and easy way to find out about soil–cement and compare it to unconfined compressive strength based on the linear relationship shown in Eq. (5).

## Data Availability

The datasets used and/or analysed during the current study available from the corresponding author on reasonable request.
